# Identification and validation of a novel senescence-related biomarker for thyroid cancer to predict the prognosis and immunotherapy

**DOI:** 10.3389/fimmu.2023.1128390

**Published:** 2023-01-24

**Authors:** Yangyang Guo, Kenan Cen, Qiaoqiao Chen, Ying Dai, Yifeng Mai, Kai Hong

**Affiliations:** ^1^ Department of Thyroid and Breast Surgery, Ningbo First Hospital, Ningbo, Zhejiang, China; ^2^ Department of Thyroid and Breast Surgery, Ningbo Hospital of Zhejiang University, Ningbo, Zhejiang, China; ^3^ Department of Geriatrics Medicine, The Affiliated Hospital of Medical School of Ningbo University, Ningbo, Zhejiang, China; ^4^ Reproductive Medicine Center, The Affiliated Drum Tower Hospital of Nanjing University Medical School, Nanjing, Jiangsu, China; ^5^ Key Laboratory of Reproductive Dysfunction Management of Zhejiang Province Assisted Reproduction Unit, Department of Obstetrics and Gynecology, Sir Run Run Shaw Hospital, Zhejiang University School of Medicine, Hangzhou, Zhejiang, China

**Keywords:** thyroid cancer, cellular senescence, tumor microenvironment, signature, immunotherapy, prognosis

## Abstract

**Introduction:**

Cellular senescence is a hallmark of tumors and has potential for cancer therapy. Cellular senescence of tumor cells plays a role in tumor progression, and patient prognosis is related to the tumor microenvironment (TME). This study aimed to explore the predictive value of senescence-related genes in thyroid cancer (THCA) and their relationship with the TME.

**Methods:**

Senescence-related genes were identified from the Molecular Signatures Database and used to conduct consensus clustering across TCGA-THCA. Differentially expressed genes (DEGs) were identified between the clusters used to perform multivariate Cox regression and least absolute shrinkage and selection operator regression (LASSO) analyses to construct a senescence-related signature. TCGA dataset was randomly divided into training and test datasets to verify the prognostic ability of the signature. Subsequently, the immune cell infiltration pattern, immunotherapy response, and drug sensitivity of the two subtypes were analyzed. Finally, the expression of signature genes was detected across TCGA-THCA and GSE33630 datasets, and further validated by RT-qPCR.

**Results:**

Three senescence clusters were identified based on the expression of 432 senescence-related genes. Then, 23 prognostic DEGs were identified in TCGA dataset. The signature, composed of six genes, showed a significant relationship with survival, immune cell infiltration, clinical characteristics, immune checkpoints, immunotherapy response, and drug sensitivity. Low-risk THCA shows a better prognosis and higher immunotherapy response than high-risk THCA. A nomogram with perfect stability constructed using signature and clinical characteristics can predict the survival of each patient. The validation part demonstrated that ADAMTSL4, DOCK6, FAM111B, and SEMA6B were expressed at higher levels in the tumor tissue, whereas lower expression of MRPS10 and PSMB7 was observed.

**Discussion:**

In conclusion, the senescence-related signature is a promising biomarker for predicting the outcome of THCA and has the potential to guide immunotherapy.

## Introduction

Thyroid cancer (THCA) is the most common malignant disease of the endocrine system, and its incidence has steadily increased in recent years (1). Among THCA, 90% of cancers are epithelial cell-derived, which are then divided into papillary thyroid cancer (PTC), follicular thyroid cancer (FTC), and anaplastic thyroid cancer (ATC). In addition, less than 5% of THCA cases are diagnosed as medullary thyroid cancer (MTC) (2). In 2022, statistics revealed 11, 860 and 31, 940 new cases of THCA in American men and women, respectively (3). Despite the low mortality, some cases may progress to invasive diseases, and recurrence and metastasis occurs in approximately 10–30% of patients (4). Thus, aggressive THCA may benefit from immunotherapy and targeted treatments.

Cellular senescence responds to diverse intrinsic and extrinsic stimulation to remove senescent cells and maintain homeostasis (5). Cellular senescence can be caused by mitosis, carcinogenic activation, tissue damage signaling, progressive telomere shortening, oxidation, genotoxic stress, telomere structure change, ionizing radiation, epigenetic changes, chromatin disorders, protein steady-state disorders, mitochondrial dysfunction, inflammation, radiation therapy, or chemotherapy (6). Accumulation of cell damage leads to both cell senescence and cancer. Cell senescence and cancer are closely associated. Evidence has shown that senescence is both beneficial and harmful to tumorigenesis and cancer progression. Senescence causes cells to remain in a permanent cell stagnation cycle, which can prevent tumor formation. Conversely, if senescent cells cannot be eliminated in time and accumulate, they may cause tumorigenesis, invasion, progression, and metastasis (7).

The genomic profile of cancer has been widely studied in recent years. In THCA, the BRAF^V600E^ mutation is the most frequent somatic mutation site. The BRAF^V600E^ mutation has been confirmed as an independent factor influencing the radioiodine avidity of PTC with lung metastases (8). Evidence has demonstrated that a single BRAF^V600F^ mutation is not related to the prognosis of THCA; however, cooperation with other factors may lead to poor THCA outcomes. Zerfaoui et al. reported that the nuclear interaction of the Arp2/3 complex and BRAF^V600E^ leads to vemurafenib resistance and the progression of THCA (9). The thyroid gland is an organ closely associated with immunity. Hashimoto’s thyroiditis is an autoimmune disorder that has been confirmed to be a protective factor against lymph node metastasis in PTC (10). PTC is characterized by lymphocytic infiltration, which may be associated with improved prognosis (11). Therefore, evaluation of THCA genomics based on specific genes, such as ferroptosis-related, pyroptosis-related, autophagy-related, and senescence-related genes, may have significant value for predicting the prognosis and immunotherapy response.

There are various prediction models of other cancer types based on the senescence-related genes which can predict prognosis and treatment effect, demonstrating that senescence-related genes can play important roles in various cancers. We proposed that senescence-related genes can also be used to predict survival and guide therapy for THCA. To provide global evidence of senescence-related genes in thyroid cancer, we identified a senescence-related signature and demonstrated that it can reliably predict the prognosis of THCA. Functional enrichment analyses were conducted to explore putative mechanisms, and the immune cell infiltration pattern, immunotherapy response, and drug sensitivity were confirmed to be significantly related to senescence-related signatures.

## Methods and materials

### Data collection and processing

THCA mRNA expression data (FPKM) and clinical information were extracted from TCGA online database (https://portal.gdc.cancer.gov/), which included 503 tumor and 56 normal samples. A total of 432 senescence genes were identified in the MSigDB (Molecular Signatures Database) genetic database (**Supplementary Table S1**) (12). TCGA-THCA was divided into a training group and a test group at a 1:1 ratio using R software.

### Identification of the senescence clustering

To explore the correlation between the senescence-related genes, a protein-protein-interaction (PPI) network was constructed on STRING (https://string-db.org/), and the functional annotations were analyzed. To further reveal the expression patterns of senescence-related genes in THCA, consensus clustering was conducted to identify the best senescence clusters. The expression of 432 senescence genes was used to conduct the consensus clustering with the “ConsensusClusterPlus” R package (13). The consensus matrix and cumulative distribution function (CDF) were used to calculate the optimal number of clusters. To determine the survival differences between clusters, the Kaplan-Meier (K-M) method was performed between the subtypes. Subsequently, the expression difference of immune checkpoints was analyzed using the limma algorithm, and p < 0.05 was considered significantly different (14). To evaluate immune cell infiltration in the clusters, we conducted an analysis to explore different immune cell types, such as CD8 T cells, cytotoxic lymphocytes, endothelial cells, fibroblasts, monocytic lineage, myeloid dendritic cells, neutrophils, T cells, and NK cells.

### Establishment of the senescence-related signature

The limma algorithm was used to identify differentially expressed genes (DEGs) between senescence clusters (**Supplementary Table S2**). Univariate Cox regression analysis was then conducted to calculate the prognostic DEGs, with HR<1 or >1 regarded as protective or risk genes (p < 0.05). To avoid overfitting, least absolute shrinkage and selection operator (LASSO) regression analysis was performed to identify signature genes using the “glmnet” package (15). The risk score was calculated using the following formula:


$$\sum_{n}^{n}Coef\left(i\right)*Expr\left(i\right)$$

Patients with THCA were divided into low- and high-risk subgroups based on their risk score relative to the median risk score. K-M survival analysis was conducted to evaluate the prognosis of patients with low- and high-risk THCA. A receiver operating characteristic (ROC) curve was used to confirm prediction stability (16). Principal component analysis (PCA) was conducted to evaluate the separation of low- and high-risk THCA (17). To further validate the advantage and stability of the novel signature, the C-index of our signature and other three models was compared (18–20).

### Nomogram construction

To further improve the clinical value, a nomogram was constructed based on age, sex, M stage, T stage, N stage, clinical stage, and senescence-related signature (21). A calibration curve was constructed to show the relationship between the actual and predicted probabilities for the 1-, 3-, and 5-year OS. The discrimination performance of each factor for THCA was evaluated using ROC analysis.

### Clinical correlation analysis

To explore the correlation between the senescence-related signature and several clinical characteristics, subgroup analyses of the training dataset were conducted, including age, sex, T stage, M stage, N stage, and clinical stage. Moreover, the survival difference between low- and high-risk THCA in distinct clinical subgroups was evaluated using K-M survival analysis.

### Immune cell infiltration and immunotherapy response

To explore the relationship between immune cell infiltration and senescence-related signatures, immune cell infiltration was assessed using the XCELL, TIMER, QUANTISEQ, MCPCOUNTER, EPIC, CIBERSORT-ABS, and CIBERSORT algorithms with different colors (22–26). The correlation coefficient was calculated to evaluate the relationship between immune cells and the signature. The expression of immune checkpoints in low- and high-risk THCA was analyzed using the limma algorithm. Immune cells, including CD8 T cells, cytotoxic lymphocytes, endothelial cells, fibroblasts, monocytic lineage, myeloid dendritic cells, neutrophils, T cells, and NK cells, were also analyzed in low- and high-risk THCA and presented in violin plots.

To predict the immunotherapy response in two subsets, tumor immune dysfunction and exclusion (TIDE), CD274 (PD-L1, death-ligand 1), interferon-gamma (IFNG, a potent inducer of immune response), myeloid-derived suppressor cells (MDSC), and immunophenoscore (IPS) were calculated.

### Functional enrichment analysis

To explore the putative mechanisms underlying low- and high-risk THCA, Gene Ontology (GO) and Kyoto Encyclopedia of Genes and Genomes (KEGG) analyses were conducted with the DEGs in low- and high-risk THCA identified using the limma algorithm (p < 0.05) (27, 28). Gene set enrichment analysis (GSEA) was performed to analyze variations in pathway activities between low- and high-risk THCA (p < 0.05) (29). The annotated file “c2.cp.kegg.v7.5.1. symbols.gmt” was downloaded from MSigDB. Functional enrichment analyses were conducted using the “ClusterProfiler” R package (30).

### Assessment of the drug sensitivity

To identify the correlation between drug sensitivity and senescence-related signatures, the half-maximal inhibitory concentrations (IC50) of drugs were calculated using the “pRRophetic” R package (31). Wilcoxon signed-rank tests were used to compare the IC50 values of low- and high-risk THCA.

### Exploration of signature genes in databases

To further explore the expression of the six signature genes in THCA, the limma algorithm was used to calculate the mRNA difference between normal and tumor samples. TCGA-THCA and GSE33630 datasets were extracted for analysis (32).

### Cell culture and RT-qPCR

The normal thyroid cell line (Nthy ori-3-1) and cancer cell line (BCPAP) were obtained from the American Type Culture Collection (ATCC, Manassas, VA, USA), maintained in RPMI-1640 media (Gibco) with 10% fetal calf serum (Gibco), and incubated in 5% CO_2_ at 37°C.

Total RNA was extracted using the TRIzol lysis method. The RNA was then reverse transcribed into complementary DNA (cDNA) using the Hifair^®^ III One-Step RT-qPCR SYBR Green Kit (Yeasen, China). RT-qPCR was conducted using the Hieff^®^ qPCR SYBR Green Master Mix (Yeasen, China), according to the manufacturer’s instructions. The 2^−ΔΔCt^ method was used to calculate the relative gene expression levels. Primers were synthesized and designed by GenePharma (Shanghai, China) and their detailed sequences are listed in **Supplementary Table S3**. β-Actin was used as the control.

### Statistical analysis

The analysis and relevant figures were obtained using R software (version 4.1.1). The t-test was used to compare differences between the two groups. Spearman’s analysis was used to calculate correlation coefficients. Kaplan–Meier survival analyses with log-rank tests were performed to assess the significant differences in OS between the two groups. Statistical significance was set at p < 0.05.

## Results

### Identification of three senescence clusters

**Figure 1:**
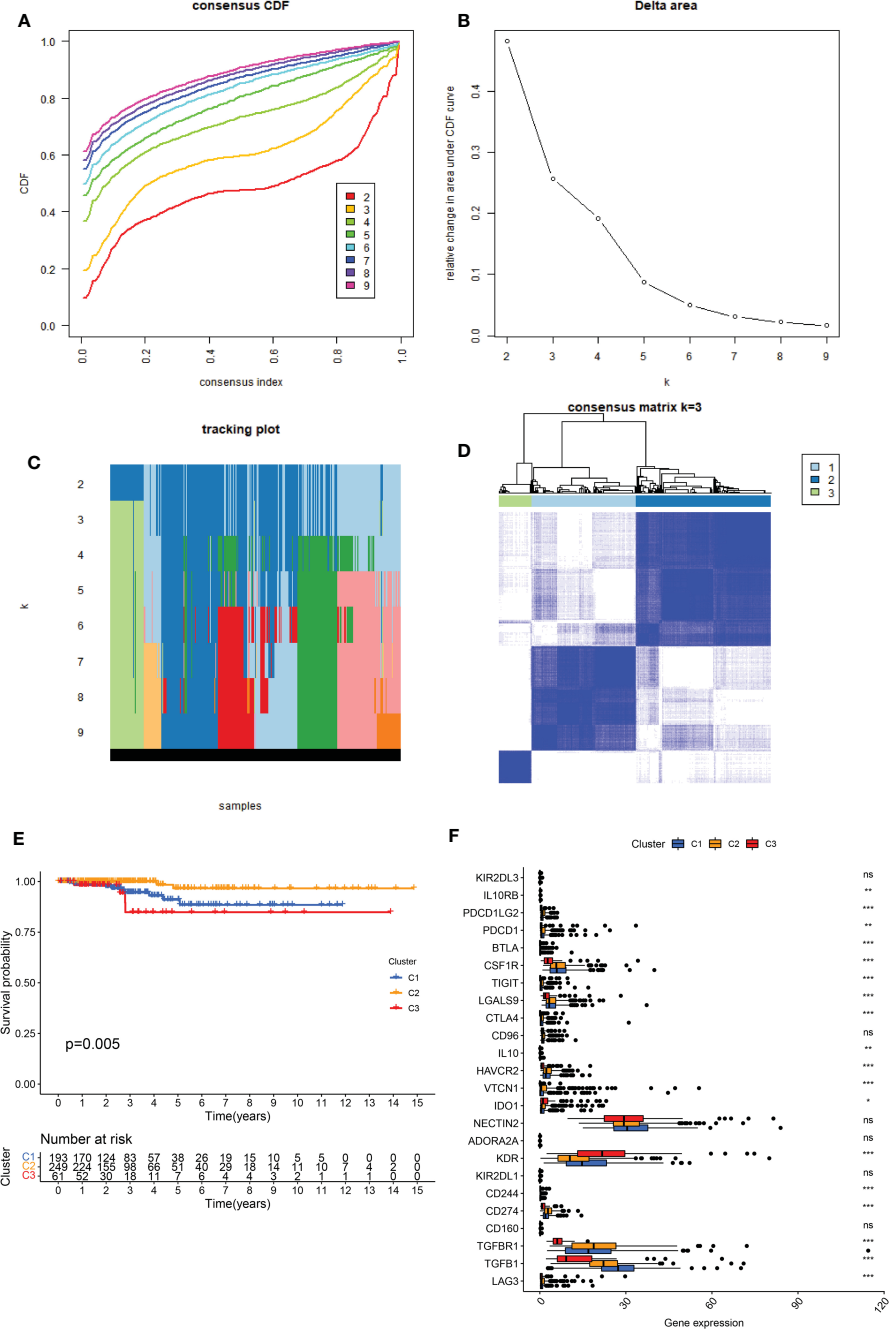
Identification of the three senescence clusters. **(A)** Consensus CDF in consistent clustering (k = 2–9). **(B)** Relative change in area under the CDF curve from k 2–9. **(C)** Tracking plot of the THCA samples (K = 2–9). **(D)** Consensus heatmap defining the three clusters (k = 3). **(E)** K-M survival analysis showing significant prognosis between the three clusters. **(F)** Boxplot presenting the significant expression difference of immune checkpoints between the three clusters. ns, no significance. * indicated P<0.05; ** indicated P<0.01; *** indicated P<0.001.

The PPI network revealed that senescence-related genes had complex correlation (**Supplementary Figure S1**) and involved in diverse cellular functions, such as organic acid metabolic process, cellular metabolic process, and metabolic process (**Supplementary File S1**). Consensus clustering results showed that there was a significant difference when k = 3 with a curve of a gentle slope (**Figures 1A-C**). Therefore, patients in TCGA-THCA were divided into clusters 1, 2, and 3. The heatmap shows that the three clusters have clear edges (**Figure 1D**). To determine whether different expression patterns of senescence-related genes affected the prognosis of THCA, K-M survival analysis was performed between the three clusters, which showed that cluster 2 had the best outcome and cluster 3 had the worst (**Figure 1E**). The relationship between senescence and immune activity was explored by analyzing the expression of immune checkpoints in the three clusters. The significantly expressed immune checkpoints included IL10RB, PDCD1LG2, PDCD1, BTLA, CSF1R, TIGIT, LGALS9, CTLA4, IL10, HAVCR2, VTCN1, IDO1, KDR, CD244, CD274, TGFBR1, TGFB1, and LAG3 (p < 0.05) (**Figure 1F**).

### Evaluation of immune cell infiltration in senescence clusters

**Figure 2:**
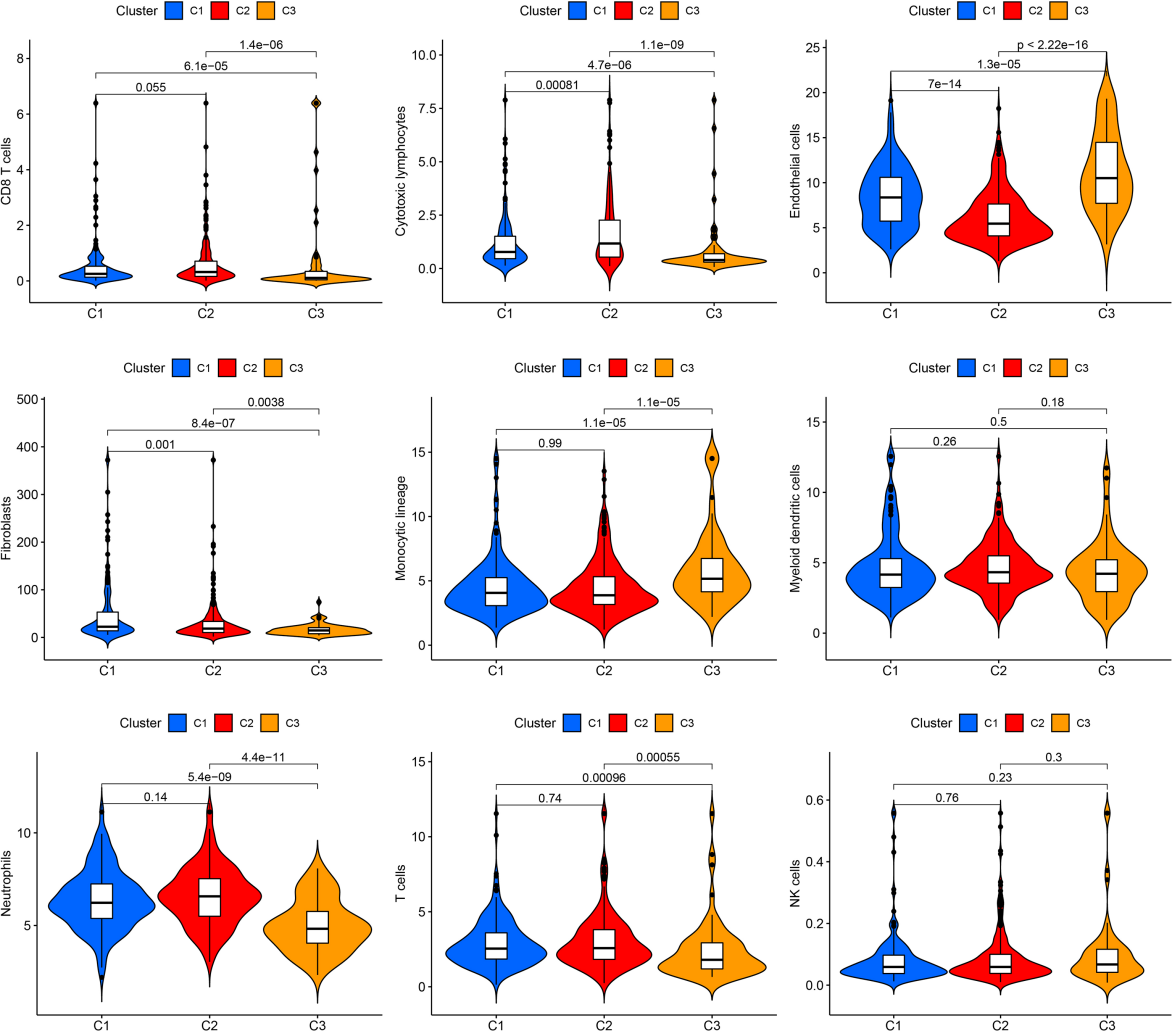
Immune cell infiltration in the three clusters. Violin plot showing CD8 T cells, cytotoxic lymphocytes, endothelial cells, fibroblasts, monocytic lineage, myeloid dendritic cells, neutrophils, T cells, and NK cells.

The MCPCOUNTER algorithm was used to explore immune cell infiltration in the three clusters (**Figure 2**). Cluster 1 contained the highest number of fibroblasts. Cluster 2 showed the highest numbers of CD8 T cells, cytotoxic lymphocytes, myeloid dendritic cells, neutrophils, and T cells. Cluster 3 had the highest number of endothelial cells and monocytic lineages. The high proportion of immune cells in cluster 2, which can suppress cancer cells, may partly account for the favorable prognosis.

### Identification of the senescence-related signature

**Figure 3:**
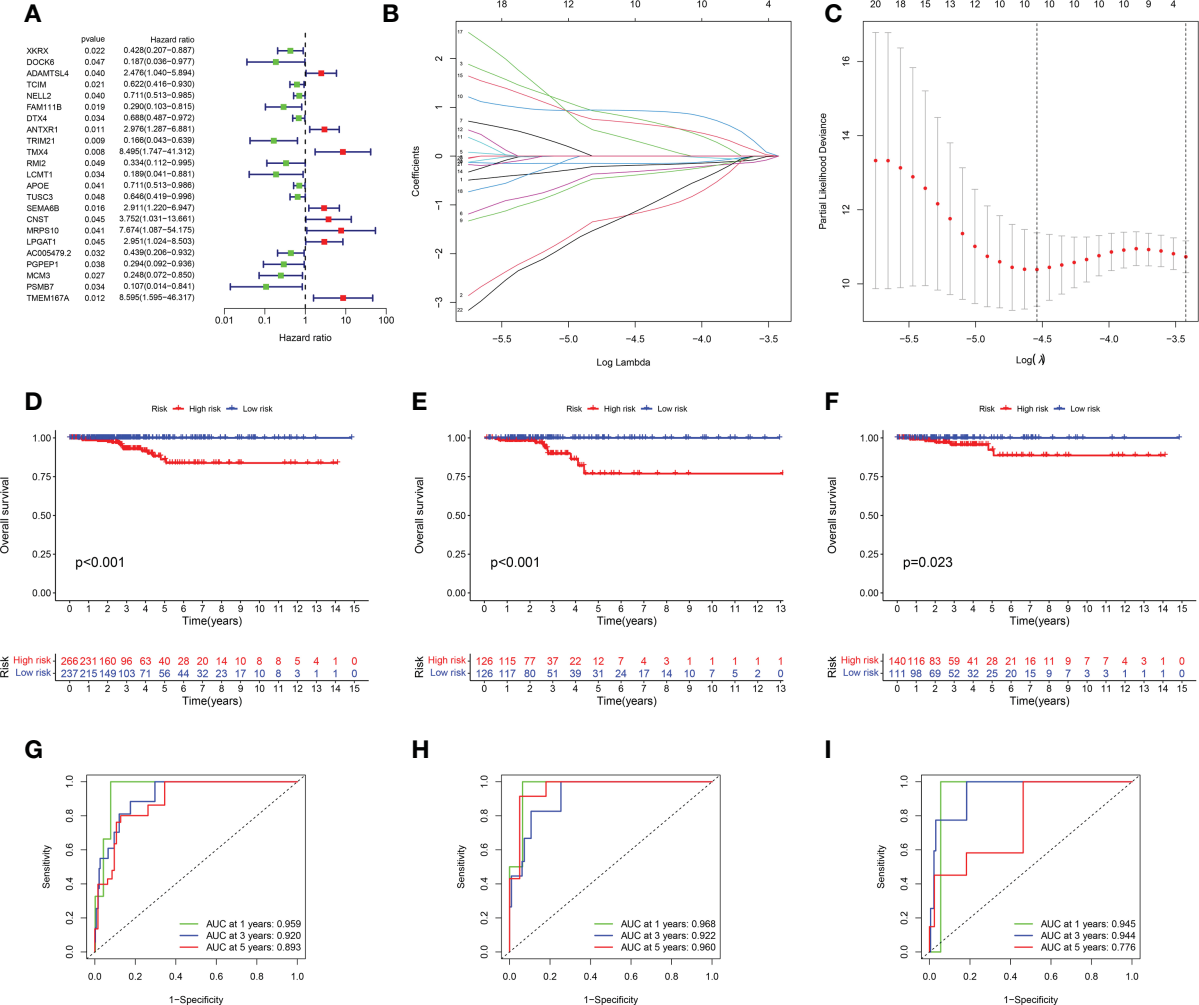
Identification and validation of the senescence-related signature. **(A)** Univariate Cox regression analysis identifying 23 prognostic DEGs. **(B)** Coefficients of the LASSO analysis. **(C)** The senescence-related signature obtained six prognostic genes with a minimum lambda value. **(D–F)** K-M survival analysis showing a significant survival difference between low- and high-risk THCA across the TCGA-all, TCGA-training, and TCGA-test subsets. **(G–I)** ROC analysis showing the stable prediction ability of the senescence-related signature across TCGA-all, TCGA-training, and TCGA-test subsets.

**Figure 4:**
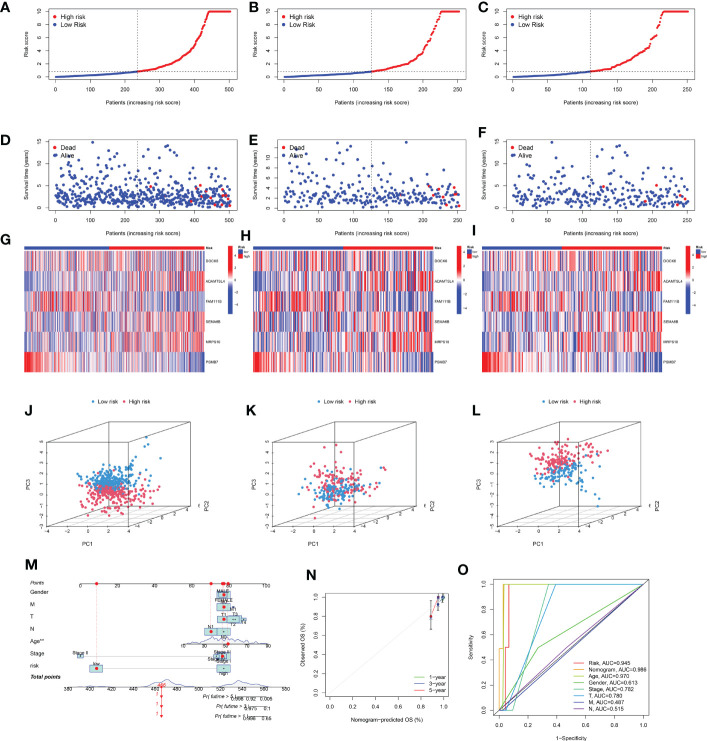
Stability of the senescence-related signature and construction of a nomogram. **(A–C)** Survival curve of the THCA patients across TCGA-all, TCGA-training, and TCGA-test subsets. **(D–F)** Survival status of the THCA patients across TCGA-all, TCGA-training, and TCGA-test subsets. **(G–I)** Heatmaps showing the expression of signature genes in THCA patients across the TCGA-all, TCGA-training, and TCGA-test subsets. **(J–L)** PCA showing the perfect separation of low- and high-risk THCA across the TCGA-all, TCGA-training, and TCGA-test subsets. **(M)** The nomogram constructed with the senescence-related signature, age, gender, T stage, M stage, N stage, and clinical stage. **(N)** The calibration curve used to estimate the prediction accuracy of the nomogram. **(O)** Multi-index ROC curve of the senescence-related signature and other factors.

Univariate Cox regression analysis was used to identify DEGs among the three senescence clusters. There were 15 protective genes, including XKRX, DOCK6, TCIM, NELL2, FAM111B, DTX4, TRIM21, RMI2, LCMT1, APOE, TUSC3, AC005479.2, PGPEP1, MCM3, and PSMB7 (Hazard Ratio, 0.428, 0.187, 0.622, 0.711, 0.290, 0.688, 0.166, 0.334, 0.189, 0.711, 0.646, 0.439, 0.294, 0.248, and 0.107, respectively), as well as eight risk genes, including ADAMTSL4, ANTXR1, TMX4, SEMA6B, CNST, MRPS10, LPGAT1, and TMEM167A (Hazard Ratio, 2.476, 2.976, 8.495, 2.911, 3.752, 7.674, 2.951, and 8,595, respectively) (**Figure 3A**). Subsequently, LASSO analysis further narrowed down the candidate genes and 10 senescence-related genes with optimal λ values were screened (**Figures 3B, C**). Six senescence-related genes were identified and used to construct the risk formula: (-1.7274663260496 * DOCK6 expression) + (1.27110457900683 * ADAMTSL4 expression) + (-0.885359668808328 * FAM111B expression) + (1.4076646152426 * SEMA6B expression) + (2.43200689265228 * MRPS10 expression) + (-4.57826818302534 * PSMB7 expression). According to the median risk score, the patients were divided into low- and high-risk subgroups. K-M survival analysis was conducted for the training subset and the two test subsets, revealing that low-risk THCA had a significantly better prognosis than high-risk THCA (p < 0.001, p < 0.001, and p = 0.023, respectively) (**Figures 3D–F**). ROC analysis showed that in TCGA-all subset the AUCs of 1-, 3-, and 5-year survival were 0.959, 0.920, and 0.893; in the TCGA-train subset, the 1-, 3-, and 5-year survival AUCs were 0.968, 0.922, and 0.960; in TCGA-test subset, the AUCs of 1-, 3-, and 5-year survival were 0.945, 0.944, and 0.776, revealing that the predictive ability of the signature was very stable (**Figures 3G–I**). Setting the median risk score as the threshold and plotting the survival status revealed that nearly all high-risk THCA patients died, further demonstrating the stability of our senescence-related signature (**Figures 4A–F**). Heatmaps showed the expression of six signature genes in low- and high-risk THCA, and the trends were consistent in the training and test subsets (**Figures 4G–I**). PCA of the three subsets confirmed that low- and high-risk THCA had perfect separation (**Figures 4J–L**). The model comparation result showed that signature of Luo et al, Li et al, and Wang et al. had lower C-index than our signature (0.589, 0.786, and 0.875 to 0.927) (**Supplementary Figure S2**).

### Construction of a nomogram

To build a more useful tool for individuals, a nomogram was constructed based on sex, M stage, T stage, N stage, age, clinical stage, and risk score (**Figure 4M**). The final nomogram scores of each patient obtained by combining all items can be used to predict 1-, 3-, and 5-year survival rates. Additionally, the calibration curves showed that the nomogram had perfect accuracy in predicting the survival (**Figure 4N**). Additionally, the ROC analysis showed that the nomogram had the highest AUC (0.986) than other factors (risk 0.945, age 0.970, gender 0.613, clinical stage 0.782, T stage 0.780, M stage 0.487, and N stage 0.515, respectively), demonstrating that the nomogram was the most stable predictive factor (**Figure 4O**).

### Clinical correlation analysis of senescence-related signature

**Figure 5:**
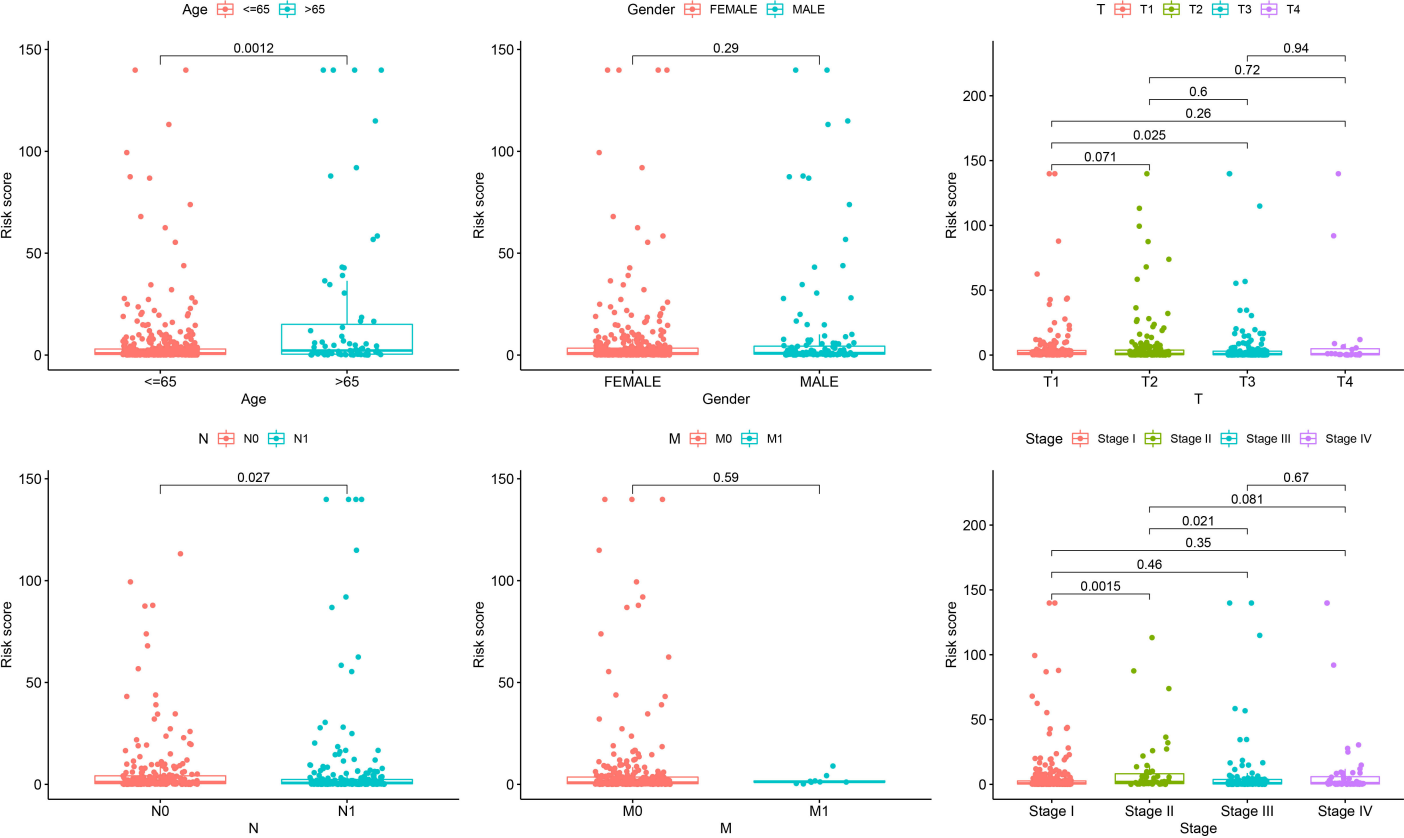
Correlation analysis showing that the senescence-related signature is associated with age, gender, T stage, N stage, M stage, and clinical stage.

To further explore the clinical correlation of the signature, the relationships between age, sex, T stage, N stage, M stage, and clinical stage and the signature were calculated. The results showed that the risks were higher in age > 65 than age ≤ 65, higher in T3 than T1, higher in N1 than N0, higher in clinical stage II than clinical stage II, and higher in clinical stage III to clinical stage II (p = 0.0012, p = 0.025, p = 0.027, p = 0.0015, and p = 0.021, respectively) (**Figure 5**).

**Figure 6:**
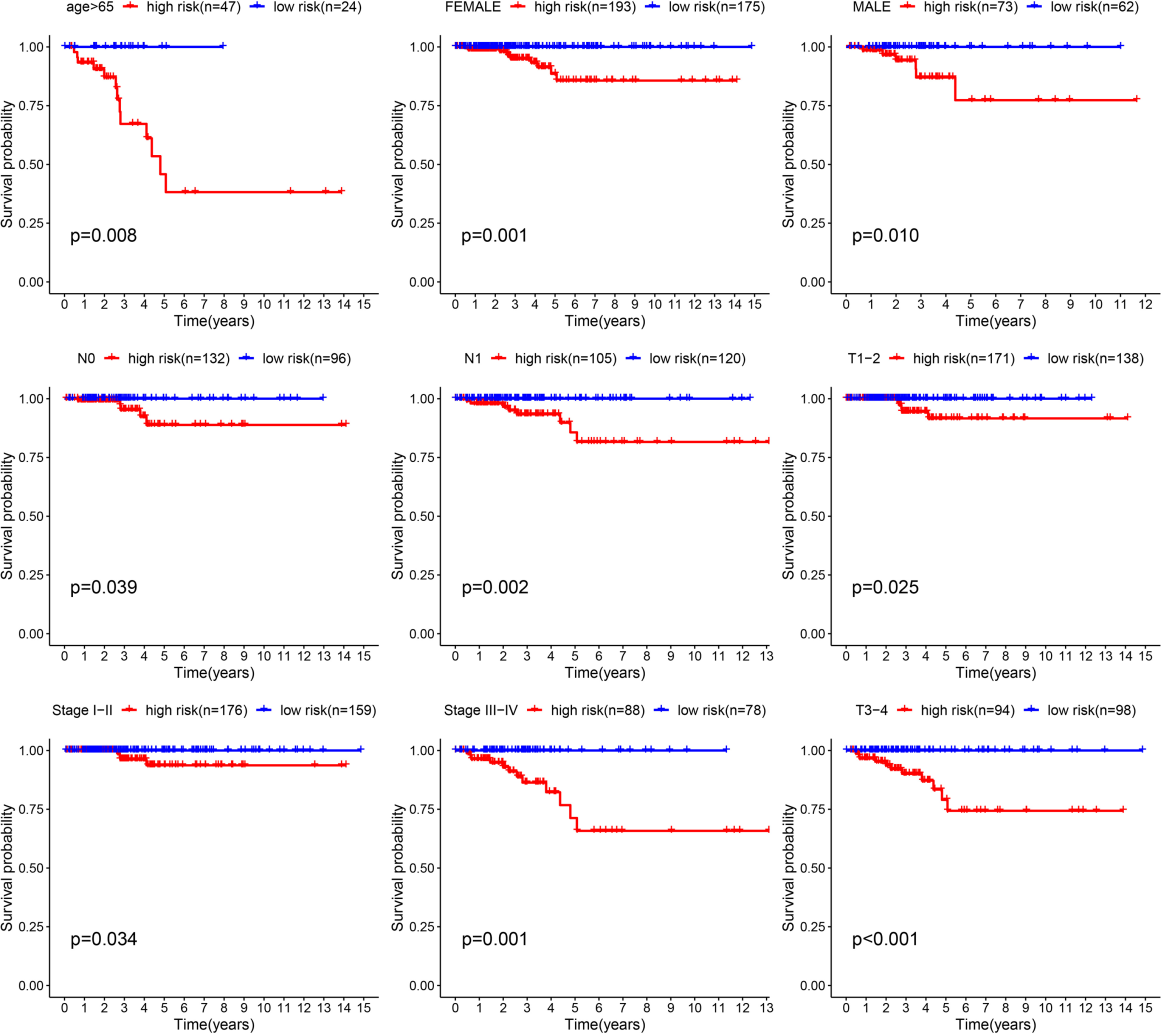
K-M survival analysis presenting the significance of prognosis between low- and high-risk THCA in subgroups of age > 65, female, male, N0, N1, T1–2, T3–4, clinical stage I–II, and clinical stage III–IV.

Although the survival difference between low- and high-risk THCA has been demonstrated in the training and test subsets, subgroup analysis was also conducted to further confirm the predictive ability of the signature. The results showed significantly better prognosis in low-risk THCA than high-risk THCA in the subgroups of age > 65 years, female, male, N0, N1, T1–2, T3–4, stage I–II, and stage III–IV (p = 0.008, p = 0.001, p = 0.010, p = 0.039, p = 0.002, p = 0.025, p = 0.001, p = 0.034, and p < 0.001, respectively) (**Figure 6**).

### Immune cell infiltration and activity

**Figure 7:**
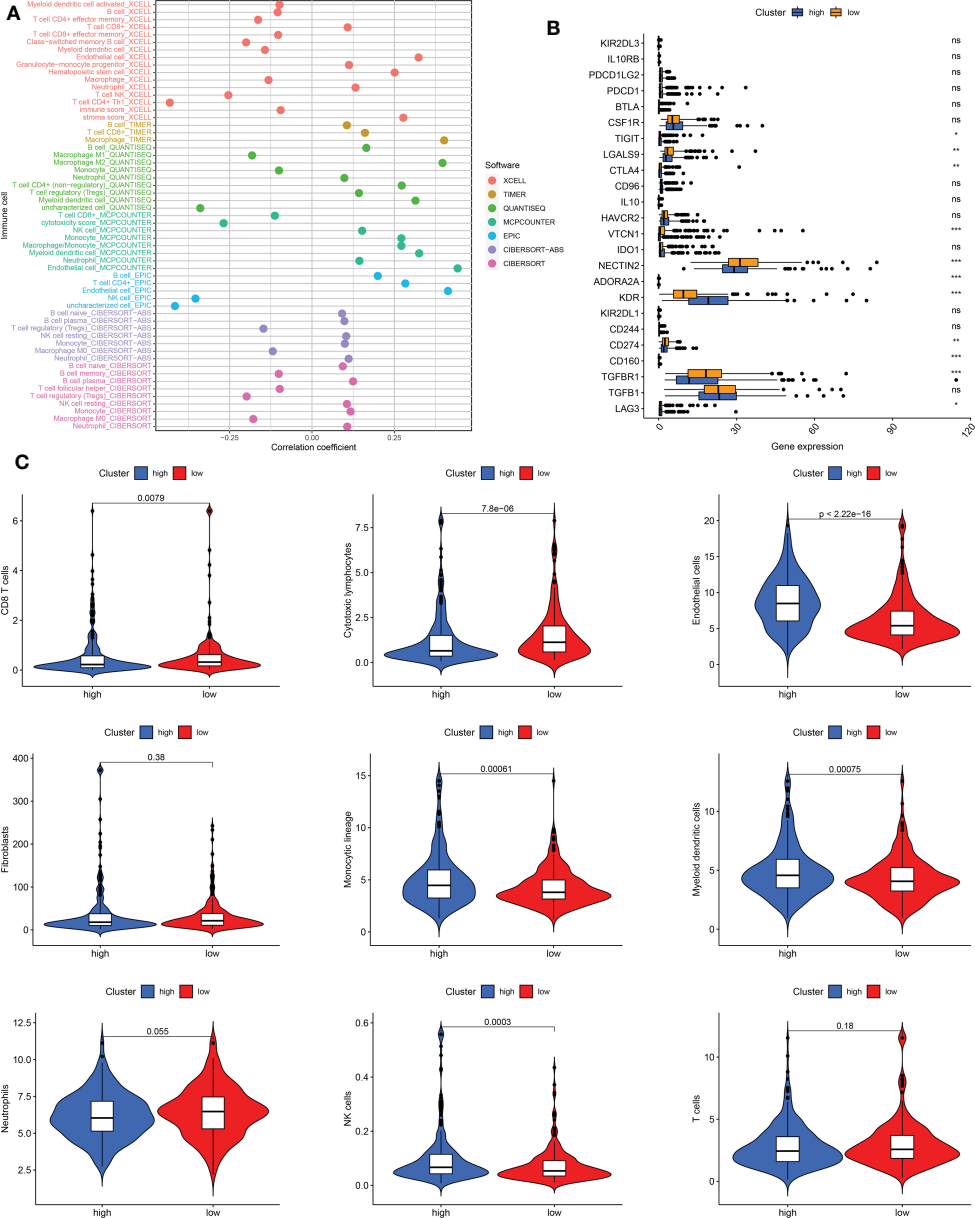
Immune cell infiltration pattern in low- and high-risk THCA. **(A)** Correlation analysis of risk score and diverse immune cells using the XCELL, TIMER, QUANTISEQ, MCPCOUNTER, EPIC, CIBERSORT-ABS, and CIBERSORT algorithms. **(B)** Boxplot showing the expression difference of immune checkpoints in low- and high-risk THCA. **(C)** Violin plot showing infiltration of CD8 T cells, cytotoxic lymphocytes, endothelial cells, fibroblasts, monocytic lineage, myeloid dendritic cells, neutrophils, NK cells, and T cells in low- and high-risk THCA. ns, no significance. * indicated P<0.05; ** indicated P<0.01; *** indicated P<0.001.

The seven immune algorithms showed that the senescence-related signature was negatively correlated with NK cells, Th1 cells, and cytotoxic cells (coefficient < -0.25), and positively correlated with endothelial cells, stromal score, macrophages, non-regulatory CD4 T cells, monocyte lineage, and myeloid dendritic cells (coefficient > 0.25) (**Figure 7A**). Regarding immune checkpoints, TIGIT, LGALS9, CTLA4, VTCN1, NECTIN2, ADORA2A, KDR, CD274, CD160, TGFBR1, and LAG3 were significantly different between low- and high-risk THCA (p < 0.01) (**Figure 7B**). There was a higher infiltration of endothelial cells, monocyte lineage, myeloid dendritic cells, and NK cells in high-risk THCA (p < 2.22e-16, p = 0.00061, p = 0.00075, and p = 0.0003, respectively). Moreover, a higher proportion of CD8 + T cells and cytotoxic lymphocytes was observed in low-risk THCA patients (p = 0.0079 and p = 7.8e-06, respectively) (**Figure 7C**).

### Immunotherapy response

**Figure 8:**
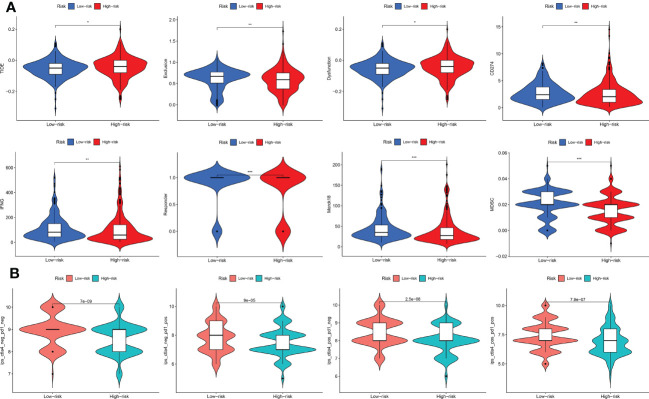
Immunotherapy response of low- and high-risk THCA. **(A)** Difference of the TIDE, Exclusion, Dysfunction, CD274, IFNG, Responder, Merck18, and MDSC score between low- and high-risk THCA. **(B)** IPS score of the low- and high-risk THCA in the of CTLA4- PD1-, CTLA4- PD1+, CTLA+ PD1-, and CTLA+ PD1+ subgroups. * indicated P<0.05; ** indicated P<0.01; *** indicated P<0.001.

The TIDE score was lower in the low-risk subtype, indicating that low-risk THCA patients may show a better response to immunotherapy (p < 0.05) (**Figure 8A**). In addition, CD274, IFNG, and MDSC levels were all higher in the low-risk subtype, which also supported a better response for low-risk THCA (p < 0.05) (**Figure 8A**). Subsequently, the IPS in the four subgroups was explored. The results showed that in the CTLA4–PD1–, CTLA4–PD1+, CTLA4+ PD1–, and CTLA4+ PD1+ subgroups, low-risk THCA exhibited a higher IPS (p = 7e-09, p = 9e-05, p = 2.5e-08, and p = 7.9e-07, respectively), which predicted a better immunotherapy response (**Figure 8B**).

### Functional enrichment analysis for low- and high-risk THCA

**Figure 9:**
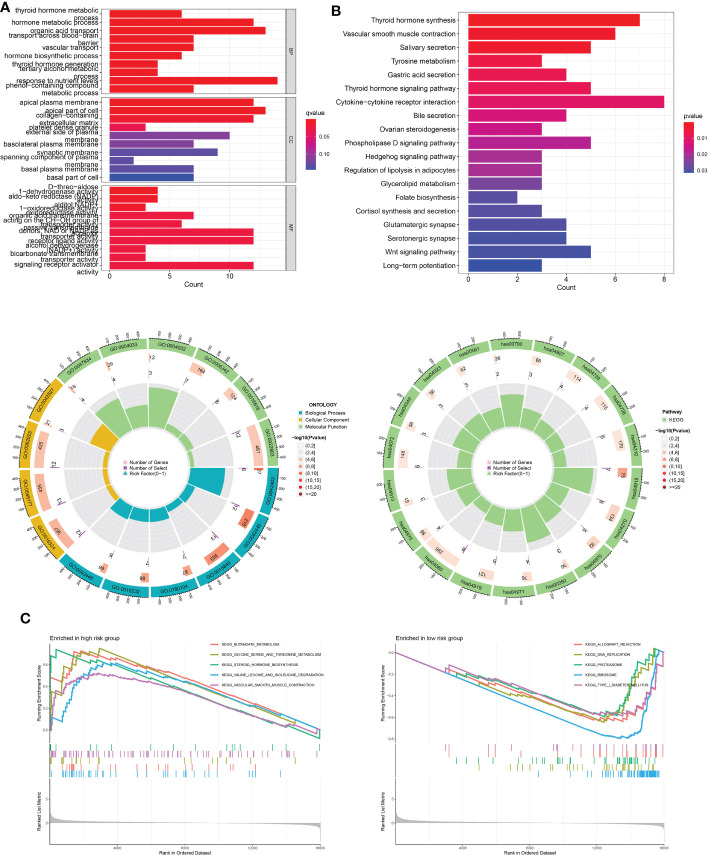
Functional enrichment analysis of low- and high-risk THCA. **(A)** GO enrichment results across TCGA-THCA including BP, CC, and MF analysis. **(B)** KEGG enrichment results showing the top related pathways across TCGA-THCA. **(C)** GSEA identifying the top five gene sets in low- and high-risk THCA.

To further investigate the putative cellular function and pathway of low- and high-risk THCA, the DEGs between the two subtypes were identified with the criteria of FDR < 0.05 and p < 0.05. BP analysis showed that the top three enriched functions were thyroid hormone metabolic processes, hormone metabolic processes, and organic acid transport (**Figure 9A**). CC analysis revealed that the top three enriched functions were apical plasma membrane, apical part of cell, and collagen-containing extracellular matrix (**Figure 9A**). MF analysis confirmed that the d-threo-aldose 1-dehydrogenase, aldo-keto reductase (NADP), and alditol NADP+ 1-oxidoreductase activities were the most enriched functions (**Figure 9A**). KEGG analysis demonstrated that the top five enriched pathways were cytokine-cytokine receptor interaction, thyroid hormone synthesis, vascular smooth muscle contraction, Wnt signaling pathway, and phospholipase D signaling pathway (**Figure 9B**). GSEA revealed differential molecular functions of the two THCA subtypes. The results showed that butanoate metabolism, glycine, serine, and threonine metabolism, steroid hormone biosynthesis, valine, leucine, and isoleucine degradation, and vascular smooth muscle contraction play vital roles in high-risk THCA (**Figure 9C**). In addition, allograft rejection, DNA replication, proteasomes, ribosomes, and type I diabetes mellitus were the top five enriched pathways in low-risk THCA (**Figure 9C**).

### Drug sensitivity in low- and high-risk THCA

**Figure 10:**
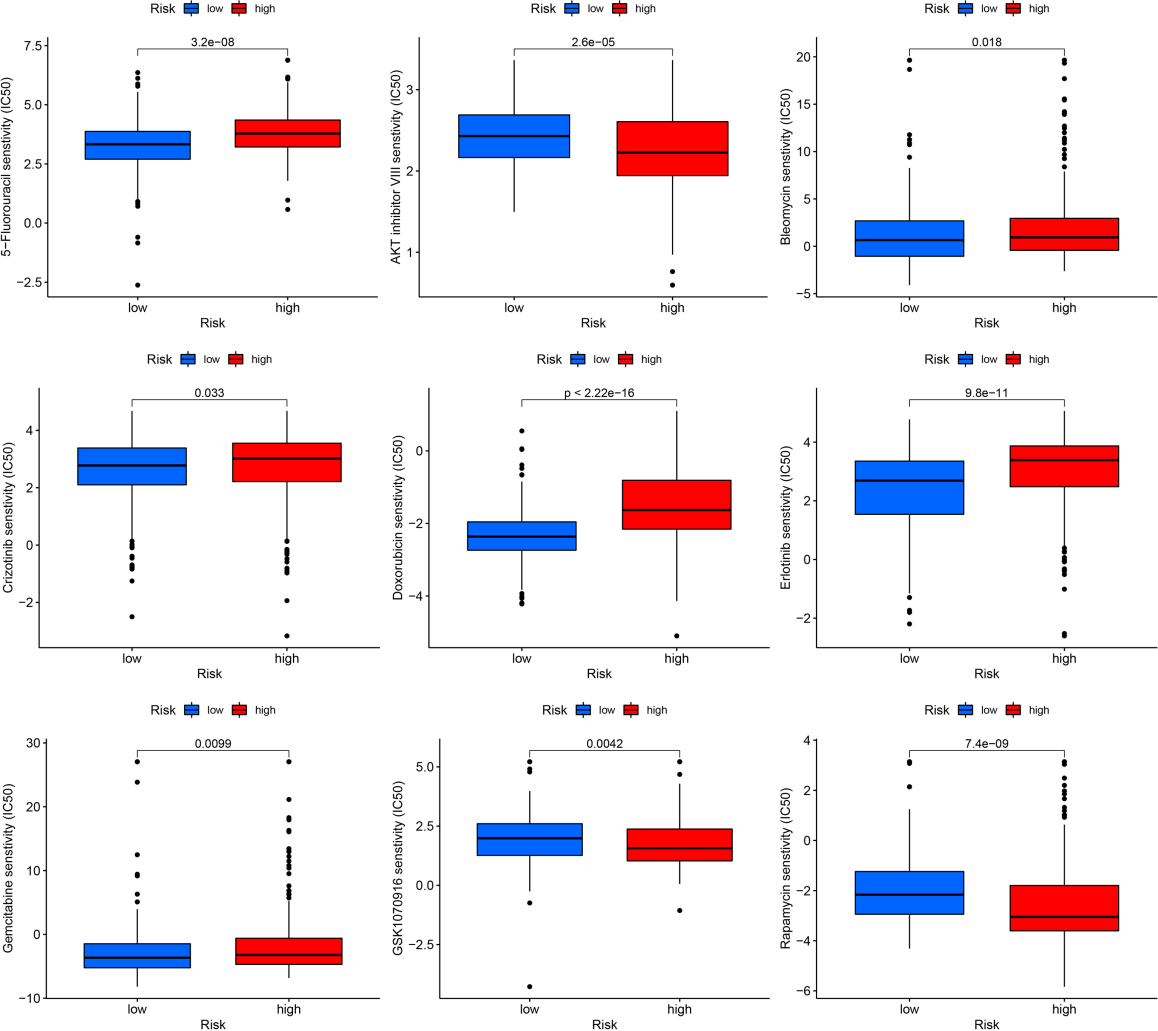
Drug sensitivity in low- and high-risk THCA, including AKT inhibitor VIII, GSK1070916, rapamycin, 5-fluorouracil, bleomycin, crizotinib, doxorubicin, erlotinib, and gemcitabine.

To predict the sensitivity of several common drugs, drug analysis was conducted for low- and high-risk THCA. AKT inhibitor VIII, GSK1070916, and rapamycin showed higher sensitivity in low-risk THCA (p = 2.6e-05, p = 0.0024, and p = 7.4e-09, respectively). Moreover, in high-risk THCA, 5-fluorouracil, bleomycin, crizotinib, doxorubicin, erlotinib, and gemcitabine were less sensitive than high-risk THCA (p = 3.2e-08, p = 0.018, and p = 0.033, p < 2.22e-16, p = 9.8e-11, p = 0.0099, respectively) (**Figure 10**).

### Expression of signature genes in THCA

**Figure 11:**
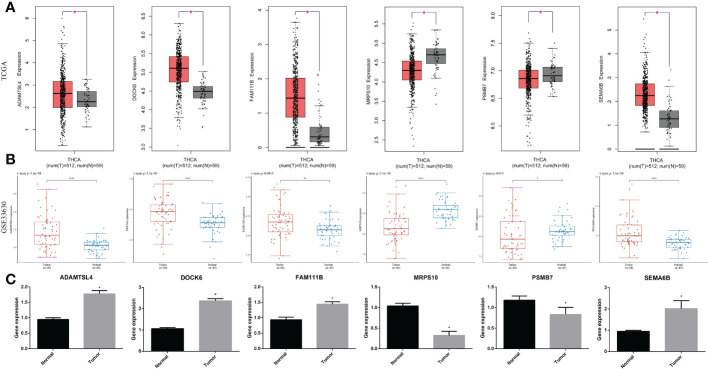
Expression of the signature gene. **(A)** Gene expression differences across TCGA dataset. **(B)** Gene expression differences across GSE33630. **(C)** RT-qPCR verifying the gene transcription in tumor and normal cells. * indicated P<0.05; ** indicated P<0.01; *** indicated P<0.001.

To further demonstrate the abnormal expression of the six signature genes in THCA, expression analysis was performed using two independent datasets, TCGA-THCA and GSE33630. The results showed that ADAMTSL4, DOCK6, FAM111B, and SEMA6B were more highly expressed in THCA than in normal samples (p < 0.05), whereas the expression of MRPS10 and PSMB7 was lower than that in normal samples (p < 0.05) (**Figures 11A, B**).

In a real-world experiment, the RT-qPCR results were consistent with the bioinformatic analysis, confirming that ADAMTSL4, DOCK6, FAM111B, and SEMA6B were expressed at higher levels in thyroid cancer cells (p < 0.05), while MRPS10 and PSMB7 were expressed at lower levels (p < 0.05) (**Figure 11C**) (**Supplementary Table S4**).

## Discussion

The cellular senescence system is complicated and multifaceted and is crucial for modulating various cellular processes. Previous studies have reported that cellular senescence has both negative and positive effects on tumorigenesis. Peng et al. demonstrated that autophagy can promote tumor suppression by inhibiting signals through senescence (33). In addition, senescence is closely related to tumor immunity. Thymic Stromal Lymphopoietin (TSLP)-stimulated CD4+ T cells play a vital role in antitumor immunity in advanced breast cancers. Boieri et al. reported that TSLP-stimulated CD4+ T cells transform breast cancer cells into a senescent-like phenotype by inducing interferon-gamma (IFN-gamma) and tumor necrosis factor-alpha (TNF-alpha) (34). Wang et al. confirmed that senescent cells could accumulate with age by expressing programmed death-ligand 1 (PD-L1) and escaping T cell immunity. PD-L1+ senescent cells showed significantly higher resistance to T-cell immunity than PD-L1- senescence cells (35). Therefore, tumor cells may escape human immunity through cellular senescence. To provide global evidence for senescence in THCA, a novel signature was identified based on senescence-related genes that could stably predict prognosis and immunotherapy response. Subgroup analysis revealed that the senescence-related signature can serve as a biomarker for the prognosis of THCA in patients aged > 65 years, females, males, N0, N1, T1–2, T3–4, clinical stage I–II, and clinical stage III–IV.

The signature comprised six genes: ADAMTSL4, DOCK6, FAM111B, SEMA6B, MRPS10, and PSMB7. Disintegrin-like and metalloproteinase domains with thrombospondin type 1 motif (ADAMTS)-like proteins are secreted glycoproteins that are part of the ADAMTS superfamily. ADAMTSL4 is one of the most widely studied members, is associated with aggressive tumor phenotypes, and participates in microfibril formation and function (36). In glioblastoma multiforme (GBM, WHO grade IV), ADAMTSL4 has been reported to make a contribution to predicting survival (37). However, the function of ADAMTSL4 in THCA requires further exploration. Dedicator of cytokinesis 6 (DOCK6) is an atypical Rho guanine nucleotide exchange factor (GEFs) for Rac and CDC42 GTPases. This is a complex protein family, and DOCK6 is one of the members of the DOCK-C subfamily that can exchange GDP for GTP for Rac1 and CDC42 (38). Previous studies have demonstrated that overexpression of DOCK6 is associated with migration and poor prognosis of oral squamous cell cancer (39). DOCK6 may promote chemotherapy and radiotherapy resistance in gastric cancer through WNT/β-catenin signaling (40). Family with sequence similarity 111 member B (FAM111B) is a 16 kb gene situated on human chromosome 11q12.1, which has shown functions in various cancer types, including thyroid cancer, pancreatic adenocarcinoma, lung cancer, and cervical cancer (41–44). Semaphorin 6b (SEMA6B) promotes and suppresses tumor progression (45). In our analysis, SEMA6B was shown to contribute to the development of THCA. Paramasivam et al. reported that gene expression screening indicates the overexpression of MRPS10 in breast cancer (46). Another study demonstrated that PSMB7 is an unfavorable prognostic marker for breast cancer and is associated with anthracycline resistance (47). The association of these genes with several types of cancer has been widely studied. The present analysis confirmed the functions of these genes in THCA.

The TME contains diverse cell types (endothelial cells, macrophages, T cells, dendritic cells, etc.) and extracellular components (extracellular matrix, cytokines, hormones, etc.) surrounding tumor cells, which affect tumor progression (48). The thyroid gland is one of the most important endocrine organs involved in human immunity. The TME of THCA is even more complicated because of the effects of other diseases, such as Hashimoto’s lymphocytic thyroiditis. Previous studies have confirmed the coexistence of Hashimoto’s disease and papillary thyroid carcinoma (49). Although some studies have reported that Hashimoto’s thyroiditis may be tumor-protective while others indicate that it is tumor-promoting, they all demonstrated that the microenvironment of the thyroid is pivotal to THCA progression (50). In the analysis of immune cell infiltration, M2 macrophages showed higher infiltration in high-risk THCA and M1 macrophages presented higher infiltration in low-risk THCA. Tumor-associated macrophages (TAMs) recruited to the microenvironment have the potential to polarize M1 or M2 macrophages according to the stimulation of TME. M1 macrophages have a pro-inflammatory role that can activate the immune response and prevent tumor progression; M2 macrophages play a completely opposite pro-tumorigenic function, both of which affect tumor development (51). M1 and M2 macrophage infiltration in low- and high-risk THCA may partly account for the differences in malignancy and prognosis. In addition, other diverse immune cell types showed a significant difference between the two subtypes, demonstrating that the TME may play a vital role in THCA.

Functional enrichment analysis revealed that various pathways play putative roles in low- and high-risk THCA, such as cytokine-cytokine receptor interactions, thyroid hormone synthesis, and the Wnt signaling pathway. Cytokine-cytokine receptor interactions have been reported to be strongly associated with the risk of diverse cancers. As an endocrine cancer, THCA has been confirmed to be affected by thyroid hormones. Moreover, after the surgery, the intake of oral L-thyroxine can both prevent the recurrence of cancer and maintain human hormones. The Wnt signaling pathway is associated with progression, drug resistance, and cancer immunotherapy (52, 53). During the past few decades, the function of Wnt signaling in THCA has also been studied. Zhang et al. reported that KDM1A could promote the progression and maintain the stemness of THCA through the Wnt signaling pathway (54). LEMD1 increases the proliferation and migration of THCA *via* the Wnt signaling pathway (55). Our analysis further demonstrated that senescence-related signatures are associated with the Wnt signaling pathway.

The bioinformatic analysis explored the issues about the prediction viability of senescence-related genes for thyroid cancer to predict the prognosis, immunotherapy response, and drug sensitivity, and discussed the putative mechanisms of senescence-related genes in thyroid cancer.

In conclusion, this study identified a novel senescence-related prognostic signature containing six genes. Comparing to other clinical gene predictive model, such as 21-gene recurrence score and 70-gene signature test (MammaPrint) for breast cancer (56, 57), the 6-gene signature has better economic viability. The risk score calculated using the signature can independently predict the survival and immunotherapy benefit of patients with THCA. Our new senescence-related model may be used for THCA-targeted therapy in the future.

## Data availability statement

The original contributions presented in the study are included in the article/**Supplementary Material**. Further inquiries can be directed to the corresponding authors.

## Author contributions

KH, QC, and YG designed the study and drafted the manuscript. KC, YD, and YM wrote the manuscript. KH, QC, and KC searched for publications and collected the data. YD and YG analyzed the data. All authors contributed to the article and approved the submitted version.
