# Corrigendum: Identification and validation of a novel senescence-related biomarker for thyroid cancer to predict the prognosis and immunotherapy

**DOI:** 10.3389/fimmu.2023.1229541

**Published:** 2023-06-05

**Authors:** Yangyang Guo, Kenan Cen, Qiaoqiao Chen, Ying Dai, Yifeng Mai, Kai Hong

**Affiliations:** ^1^ Department of Thyroid and Breast Surgery, Ningbo First Hospital, Ningbo, Zhejiang, China; ^2^ Department of Thyroid and Breast Surgery, Ningbo Hospital of Zhejiang University, Ningbo, Zhejiang, China; ^3^ Department of Geriatrics Medicine, The Affiliated Hospital of Medical School of Ningbo University, Ningbo, Zhejiang, China; ^4^ Reproductive Medicine Center, The Affiliated Drum Tower Hospital of Nanjing University Medical School, Nanjing, Jiangsu, China; ^5^ Key Laboratory of Reproductive Dysfunction Management of Zhejiang Province Assisted Reproduction Unit, Department of Obstetrics and Gynecology, Sir Run Run Shaw Hospital, Zhejiang University School of Medicine, Hangzhou, Zhejiang, China

**Keywords:** thyroid cancer, cellular senescence, tumor microenvironment, signature, immunotherapy, prognosis

In the published article, there was an error in the author list, The incorrect author list appeared as “Kai Hong ^1,2†^, Kenan Cen ^3†^,Qiaoqiao Chen ^4,5†^, Ying Dai^3†^, Yifeng Mai^3*^ and Yangyang Guo^1,2*^ “. The corrected author list appears below:

Yangyang Guo ^1,2†^, Kenan Cen^3†,^ Qiaoqiao Chen^4,5†^, Ying Dai^3†^, Yifeng Mai ^3*^ and Kai Hong^1,2*^

The authors apologize for this error and state that this does not change the scientific conclusions of the article in any way. The original article has been updated.

