# Supplementary figures and images for: Identification and validation of a novel senescence-related biomarker for thyroid cancer to predict the prognosis and immunotherapy
Source: Front Immunol. 2023 Jan 24;14:1128390. doi: 10.3389/fimmu.2023.1128390 (PMC9902917; doi:10.3389/fimmu.2023.1128390)

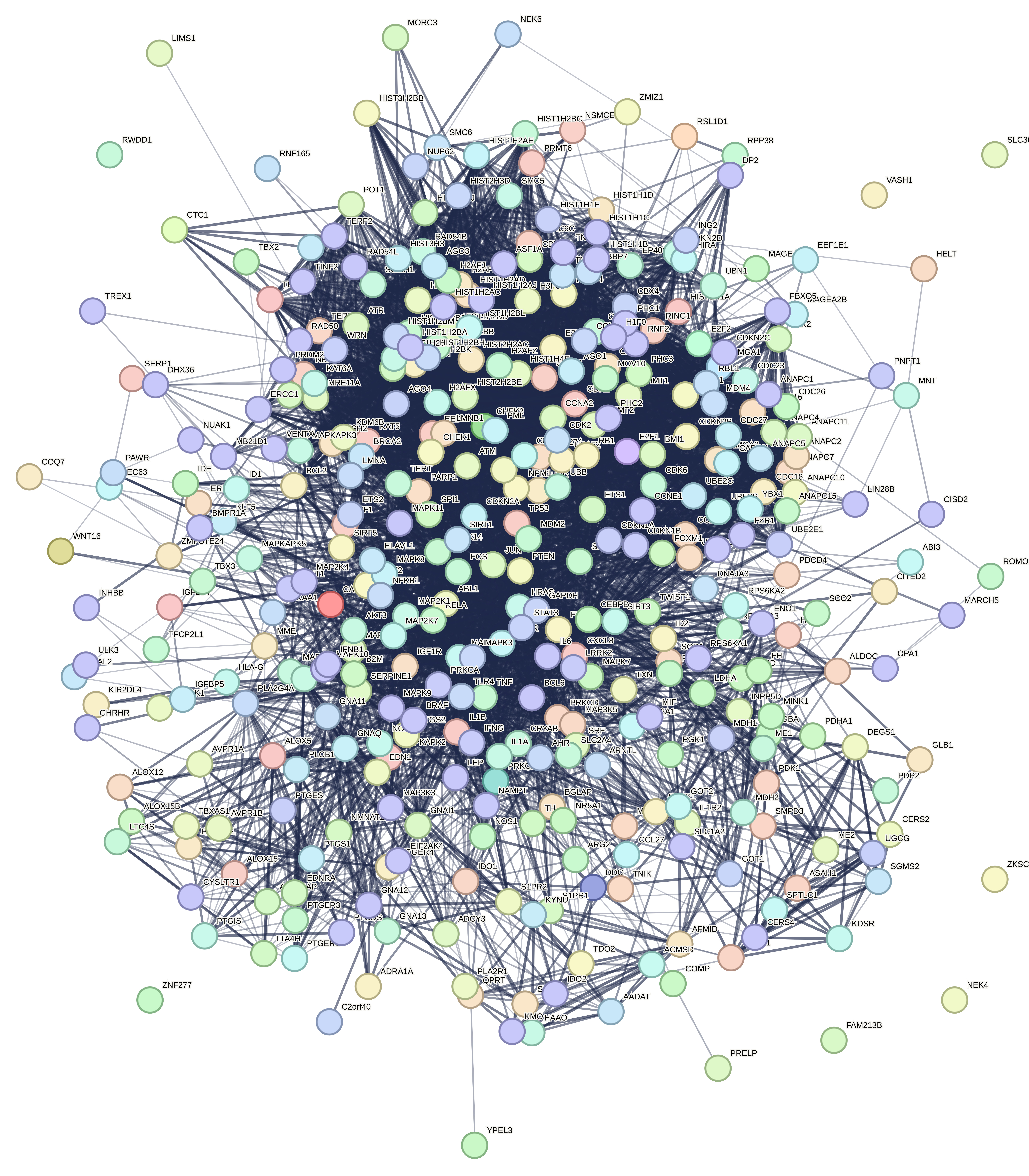

Supplement: Supplementary file 1 [file Image_1.jpeg]

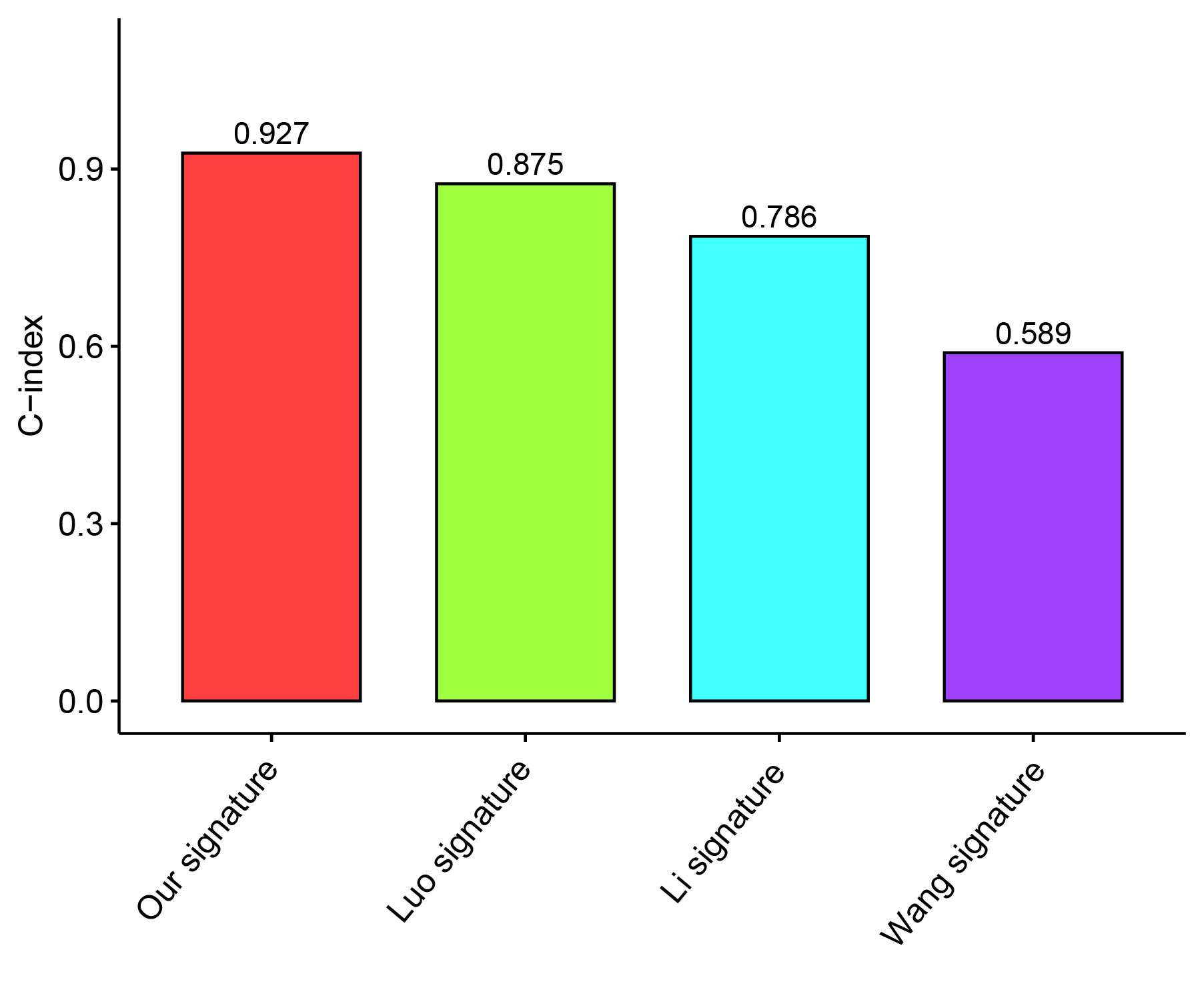

Supplement: Supplementary file 2 [file Image_2.jpeg]
